# Development and Control of Biofilms: Novel Strategies Using Natural Antimicrobials

**DOI:** 10.3390/membranes13060579

**Published:** 2023-06-02

**Authors:** Sheetal Jha, Sanjeev Anand

**Affiliations:** Dairy and Food Science Department, South Dakota State University, Brookings, SD 57007, USA

**Keywords:** biofilm, separation membrane, antimicrobial

## Abstract

Separation membranes have a wide application in the food industry, for instance, in the clarification/fractionation of milk, the concentration/separation of selected components, and wastewater treatment. They provide a large area for bacteria to attach and colonize. When a product comes into contact with a membrane, it initiates bacterial attachment/colonization and eventually forms biofilms. Several cleaning and sanitation protocols are currently utilized in the industry; however, the heavy fouling of the membrane over a prolonged duration affects the overall cleaning efficiency. In view of this, alternative approaches are being developed. Therefore, the objective of this review is to describe the novel strategies for controlling membrane biofilms such as enzyme-based cleaner, naturally produced antimicrobials of microbial origin, and preventing biofilm development using quorum interruption. Additionally, it aims to report the constitutive microflora of the membrane and the development of the predominance of resistant strains over prolonged usage. The emergence of predominance could be associated with several factors, of which, the release of antimicrobial peptides by selective strains is a prominent factor. Therefore, naturally produced antimicrobials of microbial origin could thus provide a promising approach to control biofilms. Such an intervention strategy could be implemented by developing a bio-sanitizer exhibiting antimicrobial activity against resistant biofilms.

## 1. Introduction

Biofilms are known to be the viable and non-viable multispecies communities of microorganisms (such as bacteria, fungi, yeasts, and molds) aggregated to a surface. These biofilms are firmly embedded in an extracellular polymeric substrate (EPS) attached to a substratum [1,2,3,4,5]. The EPS may contain phospholipids, proteins, polysaccharides, teichoic acids, and other polymers [6]. The stages of biofilm formation are described well by Marchand et al., 2012. It starts from the initial adherence of cells to the surface, EPS production and irreversible attachment, maturation into a three-dimensional structure, and dispersion to its planktonic form [7,8]. In the dairy and food industry, biofilm formation on separation and concentration membranes not only hampers their performance but also affects the quality and safety of the final product. The separation membrane allows the concentration and fractionation of whey in order to obtain valuable components. Various separation techniques such as ultrafiltration (UF), microfiltration (MF), nanofiltration (NF), and reverse osmosis (RO) are being utilized in the dairy industry, based on their application and selectivity. Several whey products such as whey protein concentration, whey protein isolate, whey protein hydrolysate, and reduced lactose whey are produced with different levels of minerals, protein, and lactose [9]. The concentration of whey components by means of RO helps in reducing volume and increasing solid contents prior to further processing. RO comprises a pressure-driven filtration technique with a pore size of 0.001 microns for separation that provide a large surface area for the colonization of microbes [10,11]. The texture and composition of a membrane’s surface have an impact on microbial attachment. Several factors affect the development of biofilms such as the production of exopolysaccharides, the hydrodynamics of fluid distribution systems, and inefficient disinfection [12].

Bacterial biofilms on the filtration membranes are sometimes difficult to clean due to the multilayer spiral wound structure of the membranes. These biofilms result in the biofouling of membranes. Biofouling is considered to be one of the biggest challenges to the dairy industry as the cleaning protocols may prove ineffective. That leads to the frequent premature replacement of membranes [7]. The replacement cost of membranes may constitute 25–40% of the total cost of the membrane plants subject to the type of membranes [13]. In general, membrane fouling is affected by the interaction between the foulants and membrane, the hydrodynamics of the filtration process, and the fouling layer and foulants. Additionally, the dairy feed composition is complex as it consists of different concentrations of proteins, carbohydrates, nonprotein N compounds, minerals, compounds, microorganisms, and lactic acid [14]. Based on the deposition of the substrate, membrane fouling can be categorized as chemical binding-based, crystallization-based, and particulate based. In addition, biofouling is being recognized as widespread on reverse osmosis and nanofiltration membranes [7,15].

## 2. Constitutive Microflora

The biofilm constitutive microflora could act as a reservoir for several types of microorganisms leading to the contamination of the product [16]. Several studies reported the occurrence of multispecies biofilms on separation membranes such as RO membranes [7,17]. Some of the previous studies have also demonstrated the predominance of thermo-resistant Gram-positive bacterial species, such as spores of *Bacillus licheniformis*, on whey-processing filtration membranes [18], whereas another study reported a higher predominance of Gram-negative bacterial species and coliforms as a part of their biofilm microflora. Such outcomes could be obtained in the case of water contaminations or general plant hygiene problems. One of the studies reported the presence of multispecies biofilm on water filtration membranes consisting of *Pseudomonas*, *Aeromonas*, *Aethrobacter*, *Bacillus*, *Flavobacterium*, *Actinomycetes*, and *Corynebacterium* [19].

However, several previous investigations have shown the presence of a variety of microorganisms such as *Bacillus*, *Staphylococcus*, *Micrococcus*, *Lactobacillus*, *Lactococcus*, *Klebsiella*, *Aeromonas*, *Streptococcus*, *Pseudomonas*, *Acinetobacter*, *Cronobacter*, *Corynebacterium*, *Anoxybacillus*, *Escherichia coli*, *Firmicutes*, *Proteobacterium*, *Arthrobacter,* and *Methylobacterium* as the constitutive microflora found on dairy separation membranes. Another study reported *Klebsiella* spp. and *Bacillus* spp. as the predominant species on the separation membrane used in the dairy industry [20]. Several studies reported the persistence of *Bacillus* spp. on the dairy separation membrane and its resistance to disinfection. Pathogens such as *Listeria monocytogenes*, *Bacillus cereus*, *Yersinia enterocolitica*, *Salmonella* spp., *Campylobacter jejuni*, *Staphylococcus* spp., and *Escherichia coli* 0157:H7 [10,16] have also been reported as a part of the biofilm microflora on the separation membrane. A previous study on biofilm microflora reported that *B. cereus* constituted more than 12% of the biofilm microbial community [21].

Another study demonstrated a polyphasic approach by utilizing bacterial cultivation, a 16S rDNA clone library, and fluorescence in situ hybridization techniques. This study reported the outgrowth of alpha-proteobacteria as the biofilm microflora obtained from RO and MF separation membranes. Hassan et al., 2010, reported the presence of multispecies biofilms on whey RO membranes using the scanning electron microscopic technique in addition to employing the air-drying technique for the membrane biofilm [22].

### 2.1. Resistance of Planktonic and Biofilm Embedded Cells against Cleaning Process

During the membrane cleaning process, the reversible biofilms could be removed with ease from the surface by washing. However, the irreversible biofilms may resist typical cleaning and sanitation protocols (Table 1). The transition of the planktonic phase to the biofilm formation is regulated by several physiological and environmental triggers, such as stress, nutrient availability, and quorum sensing [8]. As the biofilms accumulate from the feed and mature, these biofilms grow larger and, finally, become detached during the rinsing process [7]. Several studies reported that the biofilm microflora attached to different surfaces were different from their planktonic counterparts in terms of their resistance toward the cleaning protocol [6,15,23,24,25]. The bacterial cells showed various transcriptional programs compared to their planktonic counterparts [26]. As per the previous studies, bacterial biofilms were found to be more resistant in comparison to the planktonic cells, sometimes even up to 1000-fold, against certain specific antibiotics. Due to the complex distribution of the biofilm microflora, microbes in the biofilm were extremely resistant to disinfectants and chemical cleaners [27]. Bacterial cells were reported to survive at extreme temperatures and pH conditions ranging from −12 °C to 110 °C and 0.5 to 13 pH, respectively [28]. Microbes embedded within biofilms are more resistant to free chlorine as compared to planktonic cells. The tolerance of a mature biofilm against chlorine is mainly due to the lower penetration power of chlorine into the matrix. Only the outer surface is affected during the process, leading to a limited effect on the bacterial community within biofilms [29]. The presence of EPS that facilitates bacterial entrapment in the biofilm matrix is also one of the main reasons behind the relative ineffectiveness of disinfectants. In addition, the biofilm maturation stages also influenced the adhered cells’ resistance [30].

### 2.2. Effectiveness of Common CIP Protocols

During whey separation, the primary deposits on the RO membrane surfaces are due to the residues of minerals and proteins. Specific cleaning protocols must be in place to properly degrade these foulants. Clean-in-place (CIP) protocols using general chemicals have sometimes been found to be ineffective in removing the adhered bacterial cells [31,32,33] leading to the formation of biofilms [34,35]. Chemicals used for the cleaning process may kill the attached cells; however, they leave biomass that later leads to cell recovery and biofilm regrowth [36]. A combination of chemical, physical, and enzymatic interactions between the foulants and the agents has been developed to detach bacterial aggregates from the membrane surface, although this combination mostly relies on the mechanical power as well as the potential of the cleaning agents [37]. The appropriate selection of several chemicals in the correct order of cleaning steps was thus considered to be the key point for effective cleaning. 

Biofilm cleaning from the separation membranes is typically achieved using the circulation of various cleaners such as surfactants, metal-chelating agents, enzymes, acids, and alkalis. The cleaning protocol is performed using more favorable physical conditions with certain time–temperature and flow combinations, without dismantling the equipment. Organic foulants are hydrolyzed and solubilized using the alkaline solution by increasing their pH [38]. Most polymeric membranes tolerate a limited pH range of 3–12. The alkaline cleaners are thus more helpful in membrane flux recovery in comparison to the acidic cleaners, due to increases in membrane charge under an alkaline environment [39]. Chelating agents, on the other hand, bind the metal ions from the complex organic molecules, leading to increased cleaning efficacy [40]. Surfactants with hydrophilic and hydrophobic groups are generally found to be semi-soluble in both organic and aqueous solvents. Anions surfactants work by interacting with whey proteins to decrease the surface tension of molecules in contact. Solubilizing macromolecules usually removes foulants in these cases by forming micelles around them [41]. An acid blend is used for soluble mineral salts, whereas formulated surfactants and caustic are used for cleaning the lipid and protein residues. Enhanced cleaning is performed by using a combination of various enzymes (such as polysaccharide hydrolyzing enzyme) by breaking the proteinaceous materials and polymeric foulants [42]. Cleaning effectiveness should, however, be evaluated by enumerating the survivor viable cells after each cleaning step and based on the residual cells on the surface. Hence, it is important to understand the resistance pattern of the biofilm-embedded cells based on the survivor viable cells against the chemical cleaning protocols used for the dairy separation membranes.

### 2.3. Emergence of Bacterial Predominance within the Biofilm Matrix after the Prolonged Use of Membranes

Separation membranes (e.g., reverse osmosis membranes) have been extensively used in the dairy and food processing industry [18] for a variety of applications such as wastewater treatment, desalination, whey protein concentration, wastewater treatment, etc. [43]. Despite having numerous benefits, these separation membranes possess a significant challenge of biofouling after a prolonged duration of use [44]. The long-term use helps develop a multispecies biofilm during the contact time of the feed with the microorganism, leading to colonization and biofilm formation [45]. As discussed earlier, multispecies biofilm constitutes numerous types of microorganisms. During this process, some organisms predominate over others within the biofilm matrix [18]. Some previous studies have also depicted the emergence of single-species predominance in biofilm matrices [46,47]. A study conducted by Verma et al. worked towards understanding the microbial interaction within the biofilm microflora by studying the emergence of predominance on an 18-month-old RO of a whey processing plant. The study identified distinct bacterial isolates of *Exiguobacterium aurantiacum*, *Acinetobacter radioresistens*, *Bacillus subtilis*, and *Bacillus licheniformis* (Table 2). Further, it reported the emergence of the predominance of *Bacillus subtilis* using the co-culturing technique with fifteen combinations of the biofilm isolates [48]. 

The microorganism population shares a competitive environment to outcompete one population over another, thereby emerging as a predominant strain in a mixed-species biofilm. There are several factors associated with the emergence of the predominance of a single species amongst the constitutive microflorae. These factors include the production of certain cell metabolites, faster growth of one microorganism over another, the secretion of a narrow and broad spectrum of toxins, surface charge, the composition of the inoculum, cell channeling, the production of bacteriocins, and the release of matrix protein-like T asA, that mainly help in providing structural integrity to *Bacillus subtilis* biofilms, etc.

The predominance of one species over another could also be associated with the action of natural selection. During natural selection, when one bacterial species encounter another, it is evident that the more competitive phenotype will predominate. Due to this reason, numerous strategies ranging from resource acquisition to adhesion and matrix processing take place. One of the common ways is the secretion of narrow- and broad-spectrum toxins in addition to antitoxins that prevent self-poisoning [47]. A previous study demonstrated the role of antimicrobial compounds produced by *B. subtilis* as the potential cause of its predominance within the membrane biofilm microflora of an 18-month-old RO membrane. In this study, the culture isolates were propagated in tryptic soy agar and further microfiltered to prepare cell-free extract (CFE). The antimicrobial potential of the CFE was later determined against the biofilm microflora using an agar well assay [49]. The results from this study revealed that the reconstituted freeze-dried cell free extract reported antimicrobial activity against most of the biofilm microflora that was isolated from the 18-month-old RO membrane. Additionally, amino acid (AA) profiling was conducted to determine the constituents of the CFE. AA profiling revealed the presence of glutamic acid (11.30%) as the major constituent of the freeze-dried CFE preparation. This corresponded to the effective bactericidal activity of the antimicrobial preparation.

Another factor that could be responsible for bacterial predominance is quorum sensing (QS). It is reported to control competitive traits such as bacteriocin release. Nutrient deprivation and cell damage provide stress reactions that upregulate the bacteriocin and antibiotics. The toxin-secreting strains are known to limit the growth of sensitive strains of bacteria at a higher density of cells. The other mode of action for detecting the competitor’s presence is due to the stressful environment created in the proximity [47,50]. The emergence of predominance in the biofilm microflora provides essential information to bridge the gap between the potential of selective species to generate resistance and the development of mature biofilms on the prolonged use of membranes. This could be vital information to identify the predominating species in the biofilm matrix. It also helps in developing a CIP strategy to limit bacterial resistance and extend the life of the separation membranes in the food industry.

## 3. Novel Strategies for the Mitigation of Membrane Biofilms

### 3.1. Bio-Cleaners; Degradation of Biofilms Using Enzyme-Based Cleaners

Biofilm cleaning or the control of membrane fouling can be conducted using various techniques, such as the pretreatment of whey and maintaining the operation conditions, for instance, backwashing, crossflow, moderate pressure, and membrane regeneration. Several studies indicated the sonication and application of electrolyzed water for cake removal or decontamination due to its easy production. Under optimum conditions, the turbulent flow may also provide better cleaning efficiency. The use of various chemical cleaners can potentially impact the environment and dairy and food sectors. Hence, it creates a need to look for an alternative approach that can be organic and biodegradable in nature. Therefore, enzymatic cleaners or bio-cleaners can act as an alternative solution as they possess an advantage over chemical cleaners due to their high efficacy and compatibility with the environment. Bio-cleaners are cleaning agents consisting of enzymes that can reduce the cleaning time. Bio-cleaners are non-corrosive so they can be easily employed on Nanofiltration and reverse osmosis membranes. Enzyme cleaners work depending upon the nature of the soiling matter to be removed [51]. A typical enzymatic cleaner contains amylases that cleave the glucose linkages in polysaccharide macromolecules that lead to the release of smaller soluble polysaccharides. These soluble polysaccharides are hence easily removed while cleaning [52]. Most of the enzymatic cleaners are produced in very large quantities of the enzyme of interest in order to make them economically accessible. 

Enzymes as cleaning agents, if used alone, are incapable of breaking biofilms, as biofilm matrices are composed of a mixture of polymers that provide a certain amount of mechanical stability to biofilms [53]. Mixing an anionic detergent with an enzyme is reported to increase its performance [54]. Surfactants with chelating agents might be added with the enzymes to penetrate the biofilm matrix [55]. Surfactants and detergents neutralize the charged colloidal particles and resuspend the particles, whereas enzymes hydrolyze the proteinaceous and glycoprotein exopolymers in which the microbes are embedded [55]. These enzyme-based formulas help improve the cleaning process due to their compatibility with a lower cleaning temperature. All the different categories of enzymatic cleaning operations provide the neutralization of cleaning effluents and biodegradability [56]. Enzymes work well with mild pH, ionic strength, and temperature without adversely affecting the membrane integrity [57]. Apart from that, these enzyme-based reactions are substrate specific. The active utilization of a combination of enzymes (such as proteases and polysaccharide-hydrolyzing enzymes) is effective for removing biofilms from membrane surfaces. 

In a study reported by Bockelmann et al., 2003, they applied a-glucosidase, b-galactosidase, and lipase to degrade EPS structures in soil particulates [58]. Scanning electron microscopy images in this study revealed the effect of enzymatic treatment as a detachment of bacterial cells from soil particles. Another study conducted by Leroy et al., 2007, was based on the effect of commercial enzymes on marine biofilms and found the application of savinase, among other enzymes, for the prevention of the adherence of bacterial cells and their effective removal [59]. The supernatant produced by a marine biofilm isolate of *Bacillus licheniformis* could disperse the bacterial biofilm [60]. In a previous study, Garcia-Fernandez et al., 2017, demonstrated the use of enzyme-based cleaners to show greater biofilm removal on RO membranes when compared with commercial enzyme-based cleaners. The study evaluated the efficacy of lactase, alkaline phosphatase, and protease for the mitigation of biofilm on diverse dairy separation membranes (reverse osmosis and ultrafiltration). After testing the enzyme-based cleaners on a mixed species biofilm developed on respective membranes under lab conditions, the findings from this study suggested the importance of designing specific enzyme-based formulations depending upon specific biofilm matrices [61].

### 3.2. Use of Antimicrobial Peptides

Antimicrobial peptides (AMPs) are known to be the small cationic molecules reported to show activity against a broad range of microorganisms. Natural antimicrobial peptides are usually prepared by means of cellular tissues in a wide range of organisms and can potentially be a good source of eth synthetic AMPs. Several AMPs have the tendency to interact with the phosphate group of the lipopolysaccharides in Gram-negative bacteria. It was reported that different AMPs have different modes of action; however, most of them exhibit strong antibiofilm properties against antibiotic-resistant bacteria [62]. They are also effective with different mechanisms of action at different phases of biofilm formation.

#### Inhibition of Biofilm Microflora Using the Natural Antimicrobials

Under any optimal conditions, separation membranes should be prevented from biofilm formation as later addressing biofilm issues. Due to this reason, clean-in-place protocols are utilized for the removal or cleaning of biofilms. Several studies have stated that after the CIP regime, binary species biofilms persist and transform as a reservoir of product contamination, leading to food product spoilage. As mentioned earlier, the biofilm microflora tends to develop resistance towards chemical cleaners and their derivatives after use for a prolonged duration. Due to this, the CIP protocol remains an ineffective membrane-cleaning process. This creates a need to formulate natural antimicrobial formulation to limit biofilm growth on separation membranes. Additionally, efforts are needed to be made to find a sanitizer that does not add to the biological or chemical oxygen demand or promote biofilm-embedded bacterial resistance. Of many other possibilities that have been studied, bacteriocins (antimicrobial molecules of exact origin) offer a promising alternative for preventing or controlling biofilm formation. Bacteriocins are antimicrobial molecules of microbial origin that can inhibit the growth of various organisms, including pathogens. 

The mechanism of action of bacteriocins involves disrupting the integrity of the cell wall that initiates pore/channel formation to inhibit nucleic acid or protein synthesis [63,64]. The mitigation of biofilm formation through pore formation involves three essential steps: the adherence of the bacteriocin to the bacterial membrane, their aggregation within the membrane, and, ultimately, the formation of channels. The last stage of channel formation leads to cell constituents’ leakage and cell death.

Various *Bacillus* species have been reported to produce bacteriocins or bacteriocin-like components with varying modes of action. Some bacteriocins produced by *Bacillus* species that possess bactericidal action are tochicin [65], lichenin [66], thuricin 439, thuricin S [67], and cerein 8A [68]. Out of these, the antimicrobial potential of cerein 8A is due to pore formation, the vascularization of protoplast, and the disintegration of cells. Bacteriocins are reported to be cationic peptides demonstrating their amphiphilic and hydrophobic properties [69]. In Gram-negative strains of bacteria, the antimicrobial peptide should cross the negatively charged outer cell wall containing lipopolysaccharides (LPS) and acidic polysaccharides in Gram-positive bacteria.

Subtilosin A (subtilisin) is another type of antimicrobial peptide produced by *Bacillus subtilis* [70]. Due to the hydrophobic nature of subtilosin A, it tends to interact with the hydrophobic core of the phospholipid bilayer in the target cell membrane. A part of the negatively charged peptide is exposed to the environment and interacts with membrane receptors. The mechanism of action of subtilosin is similar to other bacteriocins (subtilin, gallidermin, and epidermin), and it forms pores through its specific interaction with the cell membrane. Previous reports have suggested that in doses above minimum inhibitory concentration (MIC), subtilosin changes into a multimeric form leading to the leakage of cellular components such as ions [71,72].

### 3.3. Preventing Biofilm Development Using Quorum Interruption

As discussed earlier, one of the reasons for the transition of microorganisms in the planktonic phase to embedded biofilm is regulated through a mechanism known as quorum sensing (QS) [8]. QS enables cell-to-cell communication and thus involves producing, releasing, detecting, and responding to small hormone-like signal molecules known as autoinducers (AIs) [73]. These signaling molecules accumulate in the surrounding environment with a rise in cell density [74]. This process also performs a significant role in regulating various physiological processes, such as the aggregation of biofilm microflora.

Based on the types of employed AIs, the QS can be classified as AI-1, AI-2, AI-3, and AHL systems. On the other hand, autoinducer-2 (AI-2) is the signaling molecule produced by the LuxS enzyme, and, therefore, it is proposed to enable interspecies communication [75]. AHLs comprise an aliphatic acyl chain of varying lengths and a lactone ring. Several other signaling molecules were also identified, such as fatty acids by *Xanthomonas* spp., *Xylella* spp., and ketones by *Vibrio* spp., while AI-2, which is a furanose borate diester, applies to both Gram-negative and Gram-positive bacteria [74]. As previous reports have demonstrated the ability of bacteria to develop biofilms through the QS mechanism, it gives rise to a possibility that QS inhibition may represent a natural, widespread antimicrobial strategy with a significant impact on biofilm formation. In the case of separation membranes, knowledge of the cell-to-cell signaling phenomenon of bacteria can be used to mitigate biofilm formation through the identification of products acting as QS antagonists. This property helps enhance the life of the membrane during the filtration process by effectively removing resilient biofilms [36]. 

Quorum quenching (QQ) has the potential of preventing the QS systems leading to a decrease in the expression of efflux pump genes [76]. Quorum quenchers are generally the counterparts of AHLs or the compounds breaking down AHLs [77]. In another mechanism, the competitor’s presence can be detected by the stress they create in close proximity. Such ‘competition sensing’ may manifest as a response to nutrient limitation or cell damage, perhaps more reliably [47,50]. 

Quorum sensing interruption works as an alternative approach to control biofilms on various surfaces by preventing or controlling the production of extracellular polymeric substrate [66,78,79,80,81]. QS helps in controlling biofilm formation at different stages of biofilm development, which includes the initial colonization/adhesion, aggregation, and maturation of biofilms. 

The widespread application of the growth-repressive agents adds up to an evolution of “super-bugs” that can resist the traditionally used inhibitory agents and are reported to affect membrane integrity due to increased residual concentration. This ultimately emphasizes the need to develop novel strategies against pathogenic and non-pathogenic microorganisms [80]. The QS interference approaches have many advantages of lower toxicity or nontoxicity, higher anti-biofouling capability, low risk of bacterial resistance advancement, and eco-friendly substances. QS is thus a biochemical path to directly control the rate and extent of biofilm development, rather than detaching biofilms after deposition using physical or chemical methods [82]. 

## 4. Future Directions

As discussed earlier, biofilm microflora potentially develops resistance to chemical cleaners and sanitizers over a prolonged duration of use. This develops resistance to their derivatives leading to an effective CIP. Therefore, future efforts could be directed towards developing an eco-friendly natural bio-sanitizer for the effective cleaning of resilient biofilms from dairy separation membranes. Additionally, the partial purification of antimicrobial peptides using size exclusion column chromatography eliminates any impurities, and enzymes can increase their antimicrobial potential. This bio-sanitizer could be naturally degraded and will lead to effective cleaning. Several microorganisms on separation membranes contribute towards biofilm formation, through quorum sensing. This gives rise to the possibility of developing a formulation with quorum-sensing inhibitors that leads to the inhibition of cell attachment, aggregation, and biofilm maturation. Future studies using spectroscopic techniques such as nuclear magnetic resonance (NMR) and circular dichroism (CD) could help in identifying the active sites and conformation of antimicrobial peptides for target protein binding.

## 5. Conclusions

Separation membranes are widely used in the dairy industry. Membrane biofouling is one of the significant challenges in the dairy and food industry and water treatment. This paper reviews reverse osmosis membrane biofouling, the major mechanism behind fouling, the constitutive microflora, and the emergence of predominance on the separation membrane. The dairy and food industries find it challenging to deal with membrane biofilms in an effective and efficient manner. Currently, several cleaning protocols are being utilized by the industry to control biofilms on membrane surfaces. However, the heavy fouling of membranes and the inadequate effectiveness of cleaning regimes make the situation difficult. To avoid this and work towards the safety and quality of membrane-processed products, several researchers have focused on either modifying the cleaning protocols by including enzymes in their cleaning regime or utilizing other natural alternatives, such as natural antimicrobials of bacterial origin (bacteriocins) and the quorum interruption of the microflora of bacterial biofilms. The recent approach of quorum signal inhibitors would help prevent bacterial colonization ability and thus biofilm formation. This approach could thus serve as a novel opportunity to control the activity of biofilm microflora without the need to utilize chemicals, disinfectants, and antibiotics.

## Figures and Tables

**Table 1:** A typical clean-in-place protocol (CIP) used for whey reverse osmosis membrane cleaning.

Step Number	CIP Steps in Sequence	Temperature (°C)	Target pH Range	Time Duration (min)
1	Alkali rinse	50	11.0–11.5	12
2	Surfactant 1	50	11.0–11.5	30
3	Acid	50	1.9–2.3	30
4	Enzyme	50	10.5–11.0	45
5	Surfactant 2	50	11.0–11.5	10
6	Sanitizer	21.1	3.0–4.0	1

**Table 2:** Constitutive microflora isolated from used RO membrane biofilms of whey processing plants.

Age of Used RO Membranes (Months)	Biofilm Microflora	Reference/Source
2	*Staphylococcus* sp. *Micrococcus* sp. *Enterococcus* sp. *Pseudomonas* sp. *Lactobacillus* sp.	[46]
6	*Aeromonas* sp. *Bacillus* sp. *Enterococcus* sp.	[22]
8	*Staphylococcus* sp. *Bacillus* sp. *Escherichia coli* *Campylobacter* sp.	[16]
12	*Lactobacillus* sp. *Lactococcus* sp. *Coliform* *Pseudomonas* sp. *Staphylococcus* sp.	[22]
14	*Escherichia coli* *Enterococcus* sp. *Staphylococcus* sp. *Klebsiella* sp.	[46]
18	*Exiguobacterium* sp. *Acinetobacter* sp. *Bacillus licheniformis* *Bacillus* sp.	[11]

## Data Availability

Not applicable.
